# Trajectories of maternal depression and offspring psychopathology at 6 years: 2004 Pelotas cohort study

**DOI:** 10.1016/j.jad.2014.12.012

**Published:** 2015-03-15

**Authors:** Alicia Matijasevich, Joseph Murray, Peter J. Cooper, Luciana Anselmi, Aluísio J.D. Barros, Fernando C. Barros, Iná S. Santos

**Affiliations:** a Department of Preventive Medicine, School of Medicine, University of São Paulo, São Paulo, SP, Brazil; b Postgraduate Program in Epidemiology, Federal University of Pelotas, Pelotas, RS, Brazil; c Department of Psychiatry, University of Cambridge, Cambridge, UK; d School of Psychology and Clinical Language Sciences, University of Reading, Reading, UK; e Stellenbosch University, Stellenbosch, South Africa; f Post-Graduate Program in Health and Behavior, Catholic University of Pelotas, Pelotas, RS, Brazil

**Keywords:** DAWBA, development and well-being assessment, EPDS, Edinburgh Post-natal Depression Scale, LMP, last menstrual period, C-section, caesarean section, OR, odds ratio, 95% CI, 95% confidence interval., Maternal postnatal depression, Group-based modelling, Development and well-being assessment, Mental health, Children, Cohort study

## Abstract

**Background:**

Few studies have addressed the course and severity of maternal depression and its effects on child psychiatric disorders from a longitudinal perspective. This study aimed to identify longitudinal patterns of maternal depression and to evaluate whether distinct depression trajectories predict particular psychiatric disorders in offspring.

**Methods:**

Cohort of 4231 births followed-up in the city of Pelotas, Brazil. Maternal depressive symptoms were assessed with the Edinburgh Postnatal Depression Scale (EPDS) at 3, 12, 24 and 48 months and 6 years after delivery. Psychiatric disorders in 6-year-old children were evaluated through the development and well-being assessment (DAWBA) instrument. Trajectories of maternal depression were calculated using a group-based modelling approach.

**Results:**

We identified five trajectories of maternal depressive symptoms: a “low” trajectory (34.8%), a “moderate low” (40.9%), a “increasing” (9.0%), a “decreasing” (9.9%), and a “high-chronic” trajectory (5.4%). The probability of children having any psychiatric disorder, as well as both internalizing and externalizing problems, increased as we moved from the “low” to the “high-chronic” trajectory. These differences were not explained by maternal and child characteristics examined in multivariate analyses.

**Limitations:**

Data on maternal depression at 3-months was available on only a sub-sample. In addition, we had to rely on maternal report of child’s behavior alone.

**Conclusions:**

The study revealed an additive effect on child outcome of maternal depression over time. We identified a group of mothers with chronic and severe symptoms of depression throughout the first six years of the child life and for this group child psychiatric outcome was particularly compromised.

## Highlights

• Maternal postnatal depressive symptomatology was modeled using group-based approach.
• 5 maternal trajectories were identified from 3 months through 6 years postpartum.
• Offspring psychiatric disorders were assessed at age 6 years.
• Psychiatric disorders increased from the “low” to the “high-chronic” trajectory.
• We revealed an additive effect on child outcome of maternal depression over time.

## 1 Introduction

Non-psychotic depression is a common health problem among women during the childbearing years, with a typical prevalence of around 14% (O’Hara and Swain, 1996), though in some contexts the prevalence appears to be considerably higher (Cooper et al., 1999). The majority of cases of postnatal depressions are self-limiting, resolving within three to six months of onset (Cooper and Murray, 1998). However, there is evidence suggesting that for many women the first episode could trigger recurrent or chronic episodes of depressive disorder (Kumar and Robson, 1984; Watson et al., 1984).

Psychopathology in parents is a strong predictor of mental disorders in children (McLaughlin et al., 2012). Approximately 40% of children of depressed parents have one or more mental disorders (not just depression), and children with parents with antisocial personality disorder also are at increased risk for a variety of disorders (Angold and Costello, 1995). Different theoretical models about genetic and environmental effects have been proposed to explain the transmission of mental disorders between generations. Although it is likely that genetic effects account for a substantial proportion of the variance, exposure to parents who have psychopathology appears to be a significant independent risk factor.

Maternal depression after childbirth is associated with serious adverse effects on family functioning, impairing the marital relationship and increasing partner conflict (Burke, 2003). It also has an adverse effect on the mother-infant relationship and can compromise infant care (Murray et al., 1996). Offspring of depressed mothers are more likely than children of non-depressed mothers to have cognitive delay and emotional difficulties as well as internalizing and externalizing problems, with evidence that these problems persist throughout childhood and adolescence (Lovejoy et al., 2000; Maughan et al., 2007; Murray et al., 2011, 2001; Sharp et al., 1995; Sinclair and Murray, 1998).

Maternal depression commonly occurs in the context of social and family risk factors, such as low socioeconomic status, unemployment, lack of parental support, and other family stressors (Pickett and Wilkinson, 2010). The combination, sequence and interrelationship of these factors may contribute directly or indirectly to the development of mental disorders in childhood, over and above the influence of maternal depression (Barker et al., 2012; Goodman and Gotlib, 1999).

Although the consequences of chronic or recurrent maternal depression on offspring mental health are well known (Brennan et al., 2000), most studies of the impact of postnatal depression on child outcomes have addressed incompletely the course and severity of maternal depression. We identified few studies, and all of them from high-income countries, that modeled trajectories of maternal depression and studied the impact of these trajectories on child psychiatric disorders (Ashman et al., 2008; Campbell et al., 2007; Cents et al., 2013; Gross et al., 2009). Only two of these studies used a group-based modelling approach to empirically derive trajectories of maternal depression. The present study, conducted in a middle-income country, had three main objectives: (1) to identify longitudinal patterns of maternal depression between three months and 6 years postpartum using a group-based modelling approach; (2) to examine predictor variables for maternal depression trajectories; and finally (3) to evaluate whether distinct maternal depression trajectories predict particular psychiatric disorders in children at age 6 years.

## 2 Methods

### 2.1 Participants

Pelotas is located in the South of Brazil and has a population of about 340,000 inhabitants. More than 99% of all deliveries take place in hospitals. In 2004, a birth cohort study attempted to enroll all births to mothers resident in the urban area. Births were identified through daily visits to the five maternity hospitals. Mothers were interviewed soon after delivery using a structured questionnaire and their newborns were examined by trained fieldworkers under the supervision of a pediatrician. Information was obtained on demographic, socioeconomic, behavioral and biological characteristics, reproductive history, and health care utilization. The non-response rate at recruitment was below 1%. A detailed description of the methodology is given elsewhere (Santos et al., 2011). All live births (*n*=4231) were enrolled in the cohort study. Follow-up assessments were made at home at mean (SD) ages 3.0 (0.1), 11.9 (0.2), 23.9 (0.4) and 49.5 (1.7) months and at a research clinic at 6.8 (0.3) years, with follow-up rates between 90 and 96%.

### 2.2 Measures

#### 2.2.1 Maternal depressive symptoms

Repeated assessments of maternal depressive symptoms were made using the Edinburgh Postnatal Depression Scale (EPDS) (Cox et al., 1987). The EPDS was originally devised for the identification of postpartum depression disorders in clinical and research settings. The EPDS is a self-administered, 10-item scale; each item has four possible responses from 0 to 3, with a total minimum score of 0 and a maximum of 30. The scale indicates the intensity of depressive symptoms over the preceding seven days. We used a Brazilian version of the questionnaire, validated in a previous study (Santos et al., 2007). The validation study showed that a cut-off score of ≥10 identified women at risk of minor depression in our population with 82.6% (75.3–89.9%) sensitivity and 65.4% (59.8–71.1%) specificity, and EPDS ≥11 identified those at risk of moderate or severe depression with 83.8% (73.4–91.3%) sensitivity and 74.7% (69.4–79.5%) specificity. Mothers׳ scores on the EPDS correlated moderately over time (from 0.42 to 0.63). The EPDS was administered to almost all of the birth cohort mothers at each follow-up, with the exception of the three-month follow-up, where the EPDS was administered to a sub-sample of 965 mothers (all mothers whose infants were born between October 1 to December 31, 2004).

#### 2.2.2 Child’s psychiatric disorders

At the six-year follow-up children were assessed using the development and well-being assessment (DAWBA; Goodman et al., 2000), validated in the Brazilian population by Fleitlich-Bilyk and Goodman (2004). The DAWBA consists of a structured interview, as well as open-ended questions, about the presence of psychiatric symptoms. It also assesses the impact of symptoms on the child׳s life. The DAWBA is designed to generate psychiatric diagnoses according to ICD-10 (World Health Organization, 1993) and DSM-IV (American Psychiatric Association, 1994) criteria for ages 5–17 years. The DAWBA was administered by trained psychologists to mothers or caregivers. The Strengths and Difficulties Questionnaire (SDQ) was also administered at the beginning of the interview, as a psychiatric screen. Thus, SDQ scores obtained on the subscales of emotional problems, conduct problems, and hyperactivity were used to determine which sections of the DAWBA would be administered (Goodman, 2001). In our study the DAWBA included the sections for separation anxiety disorder, specific phobia, social phobia, generalized anxiety disorder, posttraumatic stress disorder, panic disorder and agoraphobia, obsessive-compulsive disorder, attention deficit hyperactivity disorder (ADHD), oppositional defiant disorder, conduct disorder, eating disorders, and tic disorders. We analyzed three broad types of child psychiatric outcomes: (1) any disorder; (2) externalizing disorders, including oppositional defiant disorder, conduct disorder and any attention deficit-hyperactivity disorder (ADHD) which comprises hyperactive, inattentive and combined sub-types and ADHD not otherwise specified; and (3) internalizing disorders, including diagnoses of anxiety and depression. Two experienced child psychiatrists reviewed all the data available for each child (including free text comments made by respondents) and decided whether to accept or overturn computer-generated diagnoses. 36% of those assessed as having any DSM-IV disorder by the computer algorithm were not given any clinician-rated diagnosis. Conversely, only 6% of those assessed as not having any disorder by the computer algorithms did receive at least one clinician-rated diagnosis.

#### 2.2.3 Covariates

Information on maternal and child variables was collected in the perinatal interview. Maternal schooling at the time of delivery was recorded as complete school years of formal education. Maternal age was recorded in complete years. Women who were single, widowed, divorced, or lived without a partner were classified as single mothers. Mothers׳ skin color was self-reported and categorized as White or Black/mixed. Parity was defined as the number of previous viable pregnancies and categorized as <2 and ≥2. Maternal depression during pregnancy was defined as “present” if the mother answered positively to the following question: "*During pregnancy, did you feel depressed or have any nervous condition*?" Women were asked when their prenatal care began (first trimester or later) and if they planned their pregnancy (yes/no). Maternal smoking behavior during pregnancy was assessed retrospectively at birth by self-report. Regular smokers were defined as those women who smoked at least one cigarette daily in any trimester of pregnancy. Any amount of alcohol intake during any trimester of pregnancy was considered as alcohol consumption during pregnancy. Type of delivery was classified as vaginal or by caesarean section.

Child variables were recorded at birth. Birthweight was measured by hospital staff with 10-g precision pediatric scales that were regularly calibrated by the research team. Estimates of gestational age were based on the last menstrual period (LMP), providing they were consistent with predicted birthweight, length, and head circumference, based on the normal curves for these parameters for each week of gestational age (Fenton, 2003). If LMP-based gestational age was unknown or inconsistent, we adopted the clinical maturity estimate based on the Dubowitz method (Dubowitz et al., 1970) which was performed on almost all newborns. Births before the 37th week of pregnancy were classified as preterm. The type of hospital admission for the newborn after birth was classified as “together with the mother” and “intensive or intermediate care”.

### 2.3 Analysis plan

#### 2.3.1 Data were analyzed in three stages

First, a semiparametric, group-based modelling approach proposed by Nagin and Tremblay (1999), Nagin (2005) was used to identify the different trajectories of depressive symptoms reported by mothers based on their EPDS scores from three-months until the six-year-follow-up. Group-based trajectory modelling is a specialized form of finite mixture modelling. The method is designed to identify rather than assume groups or clusters of individuals following similar developmental trajectories. A polynomial function is used to model the relationship between an attribute (i.e. maternal depression) and age or time (Nagin and Tremblay, 1999; Nagin, 2005; Nagin and Odgers, 2010). The models were estimated with the Stata procedure “traj” (Jones and Nagin, 2012). Valid data from at least three time points are required to estimate group-based trajectories. Overall, 83.4% completed the EPDS at least three times. Because the EPDS was administered to a sub-sample of women at the three-month follow-up, the proportion of mothers that completed the EPDS in all follow-ups was 20%. We included 3332 mothers with data from at least three follow-ups in the analyses. Individuals with missing information were not excluded from the model due to the ability of group-based trajectory modelling to handle missing data using maximum likelihood estimation (Nagin, 2005). However, because an important time period (3 months postpartum) only had EPDS data available for a sub-sample of mothers in the study, we repeated analyses using this sub-sample to check the robustness of the results.

To estimate the trajectories of maternal depression, a censored normal (cnorm) model was fitted to the data because there were a significant number of mothers at the minimum of the EPDS scale (approximately 30% of women scored less than 4 points at each follow-up). The choice of the number and shape of trajectories was based not only on the best fit of the model (maximum Bayesian information criteria, BIC) but also on the interpretability of the trajectories obtained (Nagin, 2005). In addition, the selection of the appropriate model was guided by the posterior probability scores for each trajectory group (i.e., the individual’s probability of belonging to each of the trajectory groups). According to Nagin (2005), an average probability score should be higher than 0.70 for all groups.

In a second stage of analyses, we examined the contribution of predictor variables (maternal and child’s characteristics) on maternal depression trajectory group. The groups were compared on maternal and child characteristics using analysis of variance (continuous variables) and chi-square tests (categorical variables). Multinomial logit models were estimated, relating maternal group membership to predictor variables (maternal sociodemographic and behavioral variables and child characteristics), so that the parameters defining the trajectories and the probabilities of trajectory membership were estimated jointly (Nagin, 2005).

In the third stage, we evaluated whether maternal depression trajectory group membership predicted child psychiatric disorders at age 6 years. Multivariate logistic regression analysis was used to estimate the association between any psychiatric disorder, externalizing and internalizing problems at age 6 years (binary outcomes) and trajectories of maternal depressive symptoms adjusting for potential confounding factors in separate models. We considered trajectories of maternal depressive symptoms as a proximal determinant of child psychopathology, with effects that could be confounded by distal variables. An operational definition of confounding was used, that is, variables that were associated with both the outcome and the predictor of interest, and not part of the causal chain (Rothman and Greenland, 1998). Variables were grouped and included in the adjusted analysis using a backward strategy selection. Three models were included for each outcome: unadjusted results (model 1), results adjusted for maternal characteristics (model 2) and results adjusted for model 2 variables plus child characteristics (model 3). If the significance level was below 0.20, (Maldonado and Greenland, 1993) the variable remained in the model as a potential confounder for the next level. Interaction terms between maternal trajectories of depressive symptoms and gender were tested but not introduced into the model, because they did not reach statistical significance. All analyses were performed with Stata software version 12.0 (StataCorp LP, College Station, Tex).

The study protocol and all follow-ups of the 2004 Pelotas cohort studies were approved by the Medical Ethics Committee of the Federal University of Pelotas, affiliated with the Brazilian Federal Medical Council. A signed informed consent form was used in each follow-up, after informing mothers of the study objectives.

## 3 Results

### 3.1 Attrition analysis

Of the 4231 participants constituting the original cohort, 95 died in the first six years of life and 3721 were interviewed at six years. Children’s outcome data (psychiatric disorders at 6 years) were available on 3585 children. Multiple pregnancies were excluded for the present analyses (*n*=86). A final sample of 3332 mothers and children were included in the present study (78.8% of the original cohort). Women who were included were more educated, were less likely to be single, multiparous and smokers, and were more likely to be White and to have planned their pregnancy. Included and non-included women had similar age and family income and did not differ significantly in the proportion of mothers who reported depression or alcohol consumption during pregnancy. In addition, children included in the present analyses had higher birthweight and lower frequencies of preterm birth and intermediate or intensive care hospitalization at birth than those excluded (Table 1).

### 3.2 Identification of trajectories

**Fig. 1:**
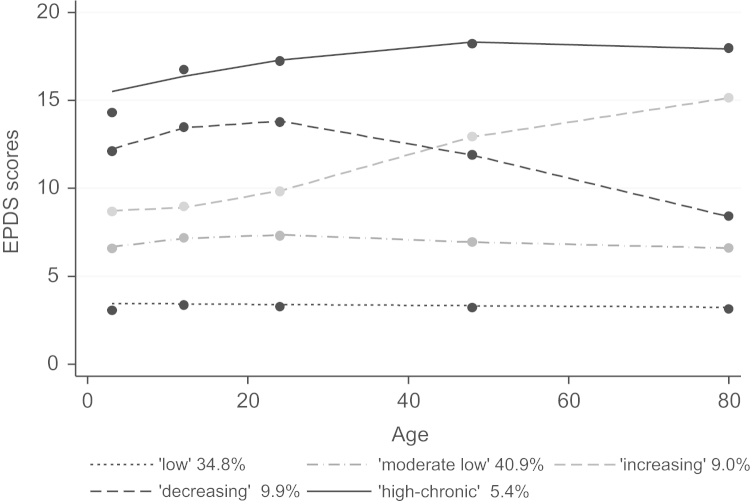
Trajectories of maternal depressive symptoms (measured by the Edinburgh Pospartum Depression Score, EPDS) by child’s age.

The first step was to model trajectories of maternal EPDS scores from the time the children were 3 months to 6 years of age. Analyses were conducted specifying three-, four-, five- and six-group models. BIC improved as more groups were added (−39790.90, −39683.64, −39536.85 and −39493.43 for three- to six-group model, respectively). The improvement observed when moving from the five-group to the six-group model was low and the five-group model emerged as the best fitting and most parsimonious model. Inspection of parameter estimates for the five-group model revealed that the constant term differed from zero for all five groups (Supplemental data file—Table S1). Three trajectories were best represented by a cubic term, one trajectory was linear and another quadratic (Fig. 1). Group 1 (named “low”, *n*=1161) and 2 (named “moderate low”, *n*=1361), representing 75.7% of the mothers, had EPDS scores <10 across all time points suggesting low depressive symptomatology. Group 3 (named “increasing”) included 9% (*n*=300) of the women in the study; these women showed a consistent increase in depressive symptoms during the study period. The fourth group (named “decreasing”), included 9.9% (*n*=329) of the women and, unlike the previous group, these mothers showed high EPDS scores in the first two years postpartum and a marked decrease afterwards. Finally, the fifth group (named “high-chronic”), which represents 5.4% of the population (*n*=181), had high EPDS scores all through the study period. For all five groups the average posterior probability was above the lower recommended threshold for assignment of 0.7 (average posterior probability of 0.87, 0.81, 0.78, 0.79 and 0.87 for Group 1 to Group 5, respectively).

Given that we had information about maternal depression at three months only for a sub-sample of around 20% of the population, as noted we performed additional analyses including only women who had EPDS at three months (*n*=877) (Supplemental data file—Fig. S1). In this sub-sample the same number of trajectories was identified with similar shape to the whole sample. However, the percentage of women in the increasing category was higher and the percentage of women in the decreasing category was correspondingly lower than in the whole sample.

### 3.3 Factors associated with maternal depression trajectory membership

The second step was to investigate whether maternal depression trajectory groups differed on maternal and child characteristics (Table 2). Mothers in the “low” depression trajectory group showed the highest incomes and schooling. In addition, they more commonly reported that their skin color was White and showed higher frequencies of early prenatal care and planned pregnancies, as well as greater frequency of births by C-section than all other groups. Mothers in the “increasing” trajectory had a higher frequency of preterm birth than those in the other groups. Women in the “high-chronic” depression trajectory group were more frequently multiparous and reported the highest rates of depression and tobacco and alcohol consumption during pregnancy. Women assigned to the “low” and “high-chronic” groups were of similar age and older than mothers in the “moderate low”, “increasing” and decreasing” trajectory groups. Finally, a higher proportion of women in both the “increasing” and “decreasing” trajectories were single mothers than women in the other groups. Regarding child characteristics, low birthweight and history of intermediate or intensive care hospitalization after birth did not vary by trajectory group.

### 3.4 Children’s outcomes at 6 years as a function of maternal depression trajectory membership

13.1% of the children (*n*=436) had a DSM-IV psychiatric disorder at six years. Internalizing problems were more frequent than externalizing problems (9.4% and 4.1%, respectively), and 7.8% of children were diagnosed with both disorders (*n*=34).

No gender interaction effect was observed for the association between maternal depression trajectory groups and psychiatric disorders at 6 years in relation to any of the three outcomes investigated (*p*=0.903. *p*=0.634 and *p*=0.620 for any psychiatric disorder, internalizing and externalizing problems, respectively).

Mothers in the “high-chronic” group had the highest prevalence of children with any psychiatric disorder as well as more children with internalizing and externalizing problems than any other group (test for linear trend *p*<0.001 for the three outcomes). Women in the “increasing” and “decreasing” trajectories reported similar levels of child psychopathology (Table 3). We performed similar analyses in the sub-sample of women with EPDS data at three months (Supplemental data file—Table S2). In this sub-sample all findings were replicated, apart from one: it was not possible to compare outcomes of any disorder or externalizing disorders between the “increasing” and “decreasing” trajectories because in the small group of children (*n*=36) whose mothers had trajectories of decreasing depression, none had externalizing disorders.

Finally, we conducted logistic regression analyses to examine differences in child psychopathology by maternal depression trajectory group after controlling for a number of potential confounders (Table 4). Parity, timing of the first prenatal visit, type of delivery, low birthweight and preterm birth were not included in any of the models because they were not associated with either psychiatric disorders or maternal trajectories. The magnitude of risk for psychiatric disorders in all maternal depressive trajectories compared to the “low” group (reference category) did not substantially change after adjusting for maternal and child characteristics. The only exception to this was observed for externalizing problems. Children of mothers in the “high-chronic” trajectory were more than ten-times as likely to have externalizing disorders in the unadjusted analysis compared to children of women in the “low” group. When maternal and child variables were taken into account, the magnitude of externalizing disorders risk decreased 37% among children of women in the “high-chronic” trajectory, while remaining almost the same as in the crude analysis among the other maternal trajectories.

## 4 Discussion

We identified five trajectories of maternal depressive symptoms from 3 months postpartum through the first 6 years of a child’s life: a “low” trajectory (34.8%), a “moderate low” trajectory (40.9%), a “increasing” (9.0%), a “decreasing” (9.9%), and a “high-chronic” trajectory (5.4%). We demonstrated that trajectory groups differed on maternal sociodemographic variables as well as on rates of preterm birth. Notably, we found that the different maternal mood trajectories were associated with different psychiatric outcomes for the children at age 6 years. The probability of children having any psychiatric disorder, as well as both internalizing and externalizing problems, increased as we moved from the “low” to the “high-chronic” trajectory. These differences were not substantially explained by the influence of maternal and child characteristics examined in multivariate analyses.

We found that there was a large proportion of mothers who reported EPDS scores below 10 (76%), indicating a low level of depressive symptoms at every follow-up from 3 months postpartum through the 6 years of their child’s life. We identified two trajectories that crossed around the 40th month after delivery, one with decreasing and the other with increasing depressive symptomatology. These two trajectories, despite their differing shapes, did not differ on either maternal and child predictor characteristics or on children psychiatric disorders at age 6 years.

Our study identified a small, although important, group of women who scored persistently high and well above the cut-off point for identifying women with probably postpartum depression (Santos et al., 2007) as well as major depression in the general population (Matijasevich et al., 2014). In line with previous literature, more than 60% of these women reported experiencing depression during pregnancy, a strong predictor of postnatal depression symptoms (Cooper et al., 1996; Milgrom et al., 2008). It is known that failure to diagnose or treat postpartum depression can have adverse long term effects on both women and their offspring (HIHCM Foundation, 2010). It is interesting to note that only 52% of women in the “high-chronic” trajectory received a medical diagnosis of depression at some point in the first four years of their child life and of these fewer than two thirds received pharmacological antidepressant treatment.

We found two previous studies that modelled trajectories of maternal depressive symptoms using a group-based modelling approach, one from mid-pregnancy to three years after childbirth (Cents et al., 2013) and the other from infant age one month to seven years (Campbell et al., 2007). Campbell et al. (2007) identified six trajectories of maternal depressive symptoms, two parallel trajectories of low and moderate symptomatology which accounted for 82% of the sample, one “moderate-increasing” and one “high-decreasing”, accounting for 6.2% and 5.6% of women respectively; a trajectory of “high-chronic” symptoms with 2.5% of mothers; and an “intermittent depression” trajectory with 3.6% of women. In our study, even when we tried a model with six trajectories, the intermittent pattern did not emerge from the data. Although the percentages of women in the other five categories were slightly different in other studies from our results, the shapes of maternal trajectories were quite similar between studies. Cents et al. (2013) identified four trajectories of maternal depressive symptoms: “no” (34%), “low” (54%), “moderate” (11%) and “high” (1.5%). Neither the “intermittent” nor the “increasing” or “decreasing” patterns appeared in their population. We identified higher percentages of women in the “high chronic” trajectory group than both previous studies. However, caution must be taken before comparing the findings of these two studies with those of our study, as they assessed maternal depression using different instruments to the one we used and they were carried out in high-income countries, where the possibility of consultation with a mental health professional and access to antidepressant medicines may well have been higher than in Brazil (Schmidt et al., 2011).

In line with previous studies, children of mothers assigned to the “high-chronic” maternal depressive symptomatology showed the highest levels of any psychiatric disorder, as well as both internalizing and externalizing problems (Campbell et al., 2007; Cents et al., 2013). It is noteworthy that it was also the case that children of mothers in the “moderate low” trajectory had more psychiatric problems than those belonging to the “low” maternal depression trajectory group. This effect has previously been observed by Cents et al. (2013), suggesting that chronic exposure to maternal depressive symptoms, even when the level of disturbance is not that high, could have harmful effects on child’s behavior (Brennan et al., 2000).

The five trajectories observed in this study differ mainly in terms of maternal characteristics. Women belonging to the “low” maternal depression trajectory group were better educated, had more financial resources and higher levels of marital stability than those assigned to the “high-chronic” trajectory. These findings are consistent with the literature showing that maternal depressive symptoms are frequently observed in a context of high levels of poverty, risk behaviors and other social stressors (Cooper and Murray, 1998), characteristics that could affect child psychopathology directly or indirectly through maternal depression. In our study, maternal trajectory membership remained a strong predictor of any child psychiatric disorder, including both internalizing and externalizing problems, after adjusting for a number of demographic, socioeconomic, behavioral and biological factors. In a recent study Barker et al. (2012) reported that even though maternal depression increased the risk of psychiatric disorders among children, multiple risk factor exposures increased the risk of both internalizing and externalizing disorders over and above the influence of maternal depression.

### 4.1 Strengths of the study

Among positive aspects, it should be pointed out that this study was a population-based sample, with longitudinal data and repeated assessments and that it used well validated instruments both for assessing maternal depressive symptomatology and child psychiatric disorders.

### 4.2 Limitations of the study

First, data on maternal depression at 3-months was available on only a sub-sample. This was unfortunate as early maternal depression could be important not only for the later child outcomes, but also as a determinant of the trajectory of maternal mood. However, we showed that similar shaped trajectories of maternal depression were observed when we limited the analyses to the sub-sample with EPDS information at three months, with only minor variations. A second limitation was that we did not have other informants regarding children’s psychiatric problems, such as teachers and fathers, and we had to rely on maternal report alone. It has been argued that measures of child behavior based on symptoms reported by depressed mothers may be affected by reporting bias (Breslau et al., 1988). However, one previous study showed that depressed mothers could be as accurate as other informants regarding their children behavior (Richters, 1992). A third limitation of our study is that causality in the association between trajectories of maternal depression and child psychopathology is difficult to infer. Previous studies have shown that ‘predictors׳ of maternal depression, such as marital conflict, could in fact represent consequences of this disorder, making it particularly difficult to identify the causal process involved (Hanington et al., 2012; Rehman et al., 2008). In addition, we could not account for genetic factors or genotype-environment interactions that may contribute significantly to the genesis of child psychiatric problems (Harold et al., 2013). Finally, our results are from a single middle-sized city and may not represent the Brazilian population as a whole.

## 5 Conclusions

In conclusion, the present study provided evidence of different trajectories of maternal postnatal depressive symptomatology and their impact on children’s psychiatric disorders. Our study revealed an additive effect on child outcome of maternal depression over time, even at low but steady levels. We identified a small but important group of mothers with chronic and severe symptoms of depression throughout the first six years of the child life and for this group child psychiatric outcome was particularly compromised. Given the potential long-term effects of maternal depression on offspring mental health, early identification, appropriate treatment and follow-up of depressed women and their children must be a key priority for mental health services.

## Authors contributions

Alicia Matijasevich participated in the design of the study, undertook the analysis, interpreted the results and drafted the first version of the article. Joseph Murray participated in the design and the analysis of the study and collaborated with the interpretation of the findings and writing of the article. Iná S. Santos participated in the design of the study and collaborated with the interpretation of the finding and writing of the article. Peter J. Cooper, Luciana Anselmi, Aluísio J. D. Barros and Fernando C. Barros collaborated with the interpretation of the findings and writing of the article. All authors approved the final version of the manuscript submitted.

## Conflict of interest

The Authors declare no conflict interests.

## Role of funding source

The funding sources had no involvement in study design; collection, analysis, or interpretation of data; in the writing of the paper; or in the decision to submit the paper for publication.

## Figures and Tables

**Table 1:** Comparison of maternal and child characteristics between those included and not included in the present study.

**Variables**	**Included (*****n*****=3332)**	**Not included (*****n*****=899)**	***p*****-Value**⁎
**Family income (reais)**, **mean (sd)**	786.5 (1065.4)	864.6 (1255.9)	0.061⁎⁎
**Schooling (years)**, **mean (sd)**	8.2 (3.4)	7.7 (3.6)	0.001⁎⁎
**Maternal age (years)**, **mean (sd)**	26.2 (6.9)	25.8 (6.6)	0.157⁎⁎
**Single mother (%)**	15.7	19.1	0.014
**Maternal skin color**, **White (%)**	73.4	71.8	0.328
**Parity**≥**2 (%)**	33.4	37.9	0.013
**Depression during pregnancy**, **(%)**	24.5	27.3	0.080
**Planned pregnancy (%)**	44.4	39.4	0.007
**Started prenatal care in the 1st trimester**, ***n*****(%)**	73.7	69.7	0.021
**Smoking during pregnancy**, ***n*****(%)**	26.7	30.3	0.035
**Alcohol during pregnancy**, ***n*****(%)**	3.2	3.7	0.494
**C-section**, ***n*****(%)**	45.1	46.6	0.423
**Child sex**, **male (%)**	52.2	50.7	0.425
**Low birthweight (%)**	7.9	18.0	<0.001
**Preterm birth (**<**37 w) (%)**	12.8	21.2	<0.001
**Intermediate or intensive care hospitalization after birth (%)**	8.3	14.4	<0.001

⁎ *x*^2^ Test.

⁎⁎ ANOVA test.

**Table 2:** Maternal and child characteristics according to maternal depression trajectory groups.

Variables	Maternal depression trajectory groups					*p*-Value⁎
	Group 1 “Low” *n*=1161	Group 2 “Moderate low” *n*=1361	Group 3 “Increasing” *n*=300	Group 4 “Decreasing” *n*=329	Group 5 “Chronic high” *n*=181	
*Maternal variables*						
Family income (reais), mean (sd)	1062.8 (1355.3)	703.3 (736.6)	620.6 (1458.5)	475.0 (466.3)	482.1 (526.5)	<0.001⁎⁎
Schooling (years), mean (sd)	9.3 (3.5)	8.1 (3.3)	6.8 (3.1)	7.3 (2.9)	5.9 (2.9)	<0.001⁎⁎
Maternal age (years), mean (sd)	27.4 (6.7)	25.5 (6.8)	25.4 (7.0)	24.4 (6.7)	27.3 (6.9)	<0.001⁎⁎
Single mother, (%)	11.5	16.2	25.3	21.0	13.8	<0.001
Skin color, White (%)	77.0	74.5	63.3	63.8	75.7	<0.001
Parity ≥2 (%)	27.3	31.5	43.7	38.9	59.7	<0.001
Depression during pregnancy (%)	5.3	29.0	34.7	43.5	61.9	<0.001
Planned pregnancy (%)	53.0	43.4	37.7	31.9	31.5	<0.001
Started prenatal care in the 1st trimester (%)	81.1	70.8	67.3	67.5	70.2	<0.001
Smoking during pregnancy (%)	16.3	28.7	36.3	35.3	47.0	<0.001
Alcohol during pregnancy (%)	2.0	3.4	2.3	2.1	13.3	<0.001
C-section (%)	50.1	42.7	46	38.0	42.5	0.001

*Child variables*						
Sex, male (%)	48.9	55.7	51.7	52.3	48.1	0.012
Low birthweight (%)	8.5	7.2	8.7	8.8	6.1	0.559
Preterm birth (<37 w) (%)	11.8	12.3	19.0	11.3	14.9	0.010
Intermediate or intensive care hospitalization after birth (%)	7.1	8.7	7.3	10.0	11.6	0.144

⁎ *x*^2^ Test.

⁎⁎ ANOVA test.

**Table 3:** Psychiatric disorders at 6 years according to maternal depression trajectory groups.

**Psychiatric disorders**	***n*****(%)**	**Maternal depression trajectory groups**					***p-Value***⁎
		Group 1 “Low” *n*=1161% (IC 95%)	Group 2 “Moderate low” *n*=1361% (IC 95%)	Group 3 “Increasing” *n*=300% (IC 95%)	Group 4 “Decreasing” *n*=329% (IC 95%)	Group 5 “Chronic high” *n*=181% (IC 95%)	
**Any psychiatric disorder**	436 (13.1)	6.2(4.9; 7.7)	12.4(10.7; 14.3)	18.7(14.4; 23.5)	20.7(16.4; 25.5)	39.2(32.1; 46.7)	<0.001
**Internalizing problems**	314(9.4)	4.9(3.7; 6.3)	8.7(7.3; 10.4)	12.7(9.1; 17.0)	15.2(11.5; 19.5)	27.6(21.3; 34.7)	<0.001
**Externalizing problems**	135(4.1)	1.3(0.7; 2.1)	3.6(2.7; 4.7)	7.3(4.7; 10.9)	7.3(4.7; 10.7)	13.8(9.1; 19.7)	<0.001

⁎ *x*^2^ Test.

**Table 4:** Crude and adjusted analyses for psychiatric disorders at 6 years according to trajectories of maternal depressive symptoms (children of mothers in the “low” depression trajectory=reference).

**Child**’**s mental health**	**Models**	**Maternal depression trajectory groups**					***p*****-Value**
		Group 1 “Low” OR (IC 95%)	Group 2 “Moderate low” OR (IC 95%)	Group 3 “Increasing” OR (IC 95%)	Group 4 “Decreasing” OR (IC 95%)	Group 5 “Chronic high” OR (IC 95%)	
**Any psychiatric disorder**	Model 1=crude analysis	1.0	2.1(1.6; 2.9)	3.5(2.4; 5.1)	3.9(2.8; 5.6)	9.8(6.7; 14.3)	<0.001
	Model 2=model 1+maternal characteristics^a^	1.0	1.9(1.4; 2.6)	3.1(2.1; 4.6)	3.4(2.3; 4.9)	8.8(5.7; 13.5)	<0.001
	Model 3=model 2+child’s variables^b^	1.0	1.9(1.4; 2.6)	3.1(2.1; 4.6)	3.3(2.3; 4.8)	8.8(5.8; 13.6)	<0.001
**Internalizing**	Model 1=crude analysis	1.0	1.9(1.4; 2.6)	2.8(1.8; 4.3)	3.5(2.3; 5.2)	7.4(4.9; 11.3)	<0.001
	Model 2=model 1+maternal characteristics^a^	1.0	1.7(1.2; 2.4)	2.6(1.7; 4.1)	3.1(2.0; 4.8)	6.9(4.3; 11.1)	<0.001
	Model 3=model 2+child’s variables^b^	1.0	1.7(1.2; 2.4)	2.6(1.7; 4.1)	3.1(2.0; 4.8)	6.9(4.3; 11.1)	<0.001
**Externalizing**	Model 1=crude analysis	1.0	2.9(1.6; 5.1)	6.0(3.1; 11.8)	6.0(3.1; 11.6)	12.2(6.3; 23.7)	<0.001
	Model 2=model 1+maternal characteristics^a^	1.0	2.3(1.2; 4.1)	4.5(2.2; 9.0)	4.1(2.1; 8.3)	8.8(4.2; 18.3)	<0.001
	Model 3=model 2+child’s variables^b^	1.0	2.1(1.2; 3.9)	4.4(2.2; 8.9)	3.9(1.9; 7.9)	8.9(4.2; 18.7)	<0.001

a Family income, schooling, age, marital status, skin color, depression and smoking and alcohol during pregnancy.

b Gender and intermediate or intensive care hospitalization after birth.
