# Supplementary material for: Trajectories of maternal depression and offspring psychopathology at 6 years: 2004 Pelotas cohort study
Source: J Affect Disord. 2015 Mar 15;174:424–31. doi: 10.1016/j.jad.2014.12.012 (PMC4351190; doi:10.1016/j.jad.2014.12.012)

Supplementary Material

Trajectories of maternal depression and offspring psychopathology at 6 years: 2004 Pelotas cohort study

Alicia Matijasevich[1,2], Joseph Murray[3], Peter J. Cooper[4,5], Luciana Anselmi[2], Aluísio J. D. Barros[2], Fernando C. Barros[2,6], Iná S. Santos[2]

[1] Department of Preventive Medicine, School of Medicine, University of São Paulo, São Paulo, Brazil

[2] Postgraduate Program in Epidemiology, Federal University of Pelotas, Pelotas, Brazil

[3] Department of Psychiatry, University of Cambridge, Cambridge, UK

[4] School of Psychology and Clinical Language Sciences, University of Reading, Reading, UK

[5] Stellenbosch University, Stellenbosch, South Africa

[6] Post-Graduate Program in Health and Behavior, Catholic University of Pelotas, Pelotas, Brazil

Supplementary Table S1.

Descriptives and average posterior probabilities (APP) for the maternal depressive symptoms trajectories (n=3332)

Trajectories of depressive symptoms	N (%)	APP (sd)	Parameters	Parameter Estimates		
				B	(se)	p-value
1 “Low” depressive symptoms	1161 (34.8)	0.87 (0.15)	Intercept	3.104	(0.146)	<0.001
			Linear	-0.005	(0.002)	0.031
2 ‘Moderate low” depressive symptoms	1361 (40.9)	0.81 (0.14)	Intercept	6.406	(0.319)	<0.001
			Linear	0.084	(0.030)	0.006
			Quadratic	-0.002	(0.001)	0.008
			Cubic	0.000	(0.000)	0.019
3 “Increasing” depressive symptoms	300 (9.0)	0.78 (0.18)	Intercept	8.950	(0.669)	<0.001
			Linear	-0.067	0.071)	0.345
			Quadratic	0.005	(0.002)	0.021
			Cubic	0.000	(0.000)	0.026
4 “Decreasing” depressive symptoms	329 (9.9)	0.79 (0.18)	Intercept	11.412	(0.760)	<0.001
			Linear	0.240	(0.077)	0.002
			Quadratic	-0.007	(0.002)	0.002
			Cubic	0.000	(0.000)	0.014
5 “High chronic” depressive symptoms	181 (5.4)	0.87 (0.17)	Intercept	15.083	(0.540)	<0.001
			Linear	0.128	(0.030)	<0.001
			Quadratic	-0.001	(0.000)	<0.001

Supplementary Table S2.

Psychiatric disorders at 6 years according to maternal depressive symptoms trajectory groups only among women with available information of the Edinburgh Postpartum Depression Score (EPDS) at three months (n=877).

Psychiatric disorders	n (%)	Maternal depressive symptoms trajectory groups					p-value *
		Group 1<br>"Low"<br>n=244<br>% (IC 95%)	Group 2<br>"Moderate low"<br>n=378<br>% (IC 95%)	Group 3<br>"Increasing"<br>n=173<br>% (IC 95%)	Group 4<br>"Decreasing"<br>n=36<br>% (IC 95%)	Group 5<br>"Chronic high"<br>n=46<br>% (IC 95%)	
Any psychiatric disorder	85 (11.0)	3.3 (1.4; 6.7)	11.2 (8.1; 15.1)	17.1 (11.5; 24.0)	9.4 (2.0; 25.0)	26.8 (14.2; 42.9)	<0.001
Internalizing problems	55 (7.1)	2.4 (0.8; 5.5)	7.1 (4.6; 10.4)	11.2 (6.7; 17.3)	9.4 (2.0; 20.0)	14.6 (5.6; 29.2)	0.005
Externalizing problems	29 (3.8)	1.0 (0.1; 3.4)	3.6 (1.8; 6.1)	6.6 (3.2; 11.8)	0.0	12.2 (4.1; 26.2)	0.002

* x^2 test

Supplementary Figure S1. Trajectories of maternal depressive symptoms (measured by the Edinburgh Postpartum Depression Score, EPDS) by child´s age only among women with available information of EPDS at three months (n=877).

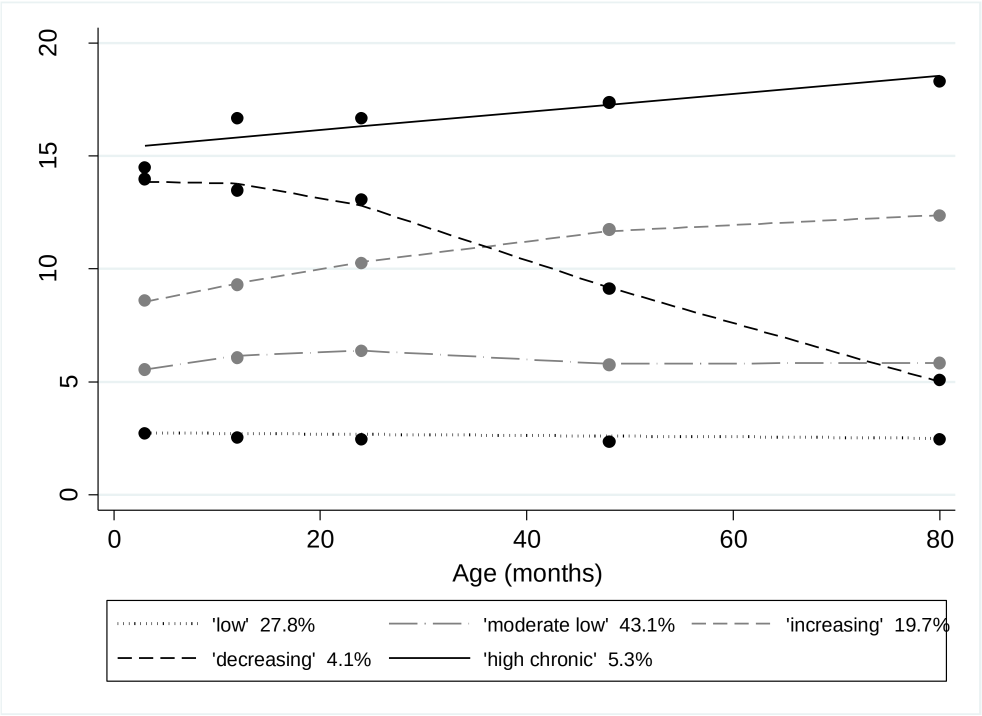

Supplement: Supplementary file 1 — Supplementary data [file mmc1.pdf]
